# Systematic discovery of novel ciliary genes through functional genomics in the zebrafish

**DOI:** 10.1242/dev.108209

**Published:** 2014-09

**Authors:** Semil P. Choksi, Deepak Babu, Doreen Lau, Xianwen Yu, Sudipto Roy

**Affiliations:** 1 Institute of Molecular and Cell Biology, 61 Biopolis Drive, Singapore138673; 2 NUS Graduate School of Integrative Sciences and Engineering, Centre for Life Sciences, 28 Medical Drive, Singapore117456; 3 Department of Biological Sciences, National University of Singapore, 14 Science Drive 4, Singapore117543; 4 Department of Paediatrics, Yong Loo Lin School of Medicine, National University of Singapore, 1E Kent Ridge Road, Singapore119288

**Keywords:** Foxj1, Motile cilia, Zebrafish, Ciliary gene screen, Ciliopathy, Primary ciliary dyskinesia

## Abstract

Cilia are microtubule-based hair-like organelles that play many important roles in development and physiology, and are implicated in a rapidly expanding spectrum of human diseases, collectively termed ciliopathies. Primary ciliary dyskinesia (PCD), one of the most prevalent of ciliopathies, arises from abnormalities in the differentiation or motility of the motile cilia. Despite their biomedical importance, a methodical functional screen for ciliary genes has not been carried out in any vertebrate at the organismal level. We sought to systematically discover novel motile cilia genes by identifying the genes induced by Foxj1, a winged-helix transcription factor that has an evolutionarily conserved role as the master regulator of motile cilia biogenesis. Unexpectedly, we find that the majority of the Foxj1-induced genes have not been associated with cilia before. To characterize these novel putative ciliary genes, we subjected 50 randomly selected candidates to a systematic functional phenotypic screen in zebrafish embryos. Remarkably, we find that over 60% are required for ciliary differentiation or function, whereas 30% of the proteins encoded by these genes localize to motile cilia. We also show that these genes regulate the proper differentiation and beating of motile cilia. This collection of Foxj1-induced genes will be invaluable for furthering our understanding of ciliary biology, and in the identification of new mutations underlying ciliary disorders in humans.

## INTRODUCTION

Cilia are highly conserved hair-like protrusions found on the surface of most cells. The core of the cilium, or the axoneme, is made up of radially arranged microtubules. The axoneme extends from the basal body, a derivative of the mother centriole, which docks with the apical cell membrane at the onset of ciliogenesis. Cilia can be broadly classified as primary and motile. Primary cilia are immotile due to a lack of axonemal dynein arms and are present on cells throughout the vertebrate body. They serve as sensory organelles for a variety of signaling pathways that function during embryonic development and in adult physiology (Goetz and Anderson, 2010). However, motile cilia, which contain dynein arms anchored to the axoneme, beat rhythmically to drive the locomotion of spermatozoa and to generate fluid flow in the embryonic node, brain ventricles, and the mammalian respiratory and reproductive tracts (Drummond, 2012; Satir and Christensen, 2007).

Given the widespread distribution and varied functions of cilia, abnormalities in these organelles underlie a large number of human genetic disorders. For example, defective motile cilia cause primary ciliary dyskinesia (PCD, MIM #244400) (Afzelius, 1976; Boon et al., 2013; Fliegauf et al., 2007). Consistent with the distribution pattern of motile cilia, symptoms of PCD include chronic respiratory infections, bronchiectasis, infertility and, in some cases, hydrocephalus. A subset of individuals with PCD also exhibits laterality defects (Kartagener syndrome) due to defective motile cilia in the embryonic node (Babu and Roy, 2013). The identification of genes regulating cilia formation and function is the key first step towards understanding the biology of cilia and ciliopathies. Despite the fact that PCD ranks among the most prevalent of ciliopathies, causative mutations in a limited number of genes have been discovered, mainly in those encoding axonemal dynein subunits (Bartoloni et al., 2002; Loges et al., 2008; Mazor et al., 2011; Olbrich et al., 2002; Pennarun et al., 1999) and dynein motor assembly proteins (Kott et al., 2012; Loges et al., 2009; Mitchison et al., 2012; Omran et al., 2008). These mutations account for fewer than two-thirds of the total cases of PCD (Zariwala et al., 2011), indicating that a large number of motile cilia genes whose mutations cause PCD have yet to be identified.

We and others have previously shown that the transcription factor Foxj1 has a master regulatory role in the biogenesis of vertebrate motile cilia (reviewed by Choksi et al., 2014; Stubbs et al., 2008; Yu et al., 2008), indicating that it coordinates the expression of a whole suite of genes that are necessary as well as sufficient for the assembly of functional motile cilia. Using genome-wide expression profiling and large-scale functional studies in the zebrafish, we now describe an extensively validated network of motile cilia genes controlled by Foxj1. We believe that this collection will be a valuable resource for understanding the basic biology of cilia and in the identification of novel mutations causative of ciliopathies such as PCD.

## RESULTS

### Foxj1-induced genes are replete with known cilia and ciliopathy genes

**Fig. 1.**
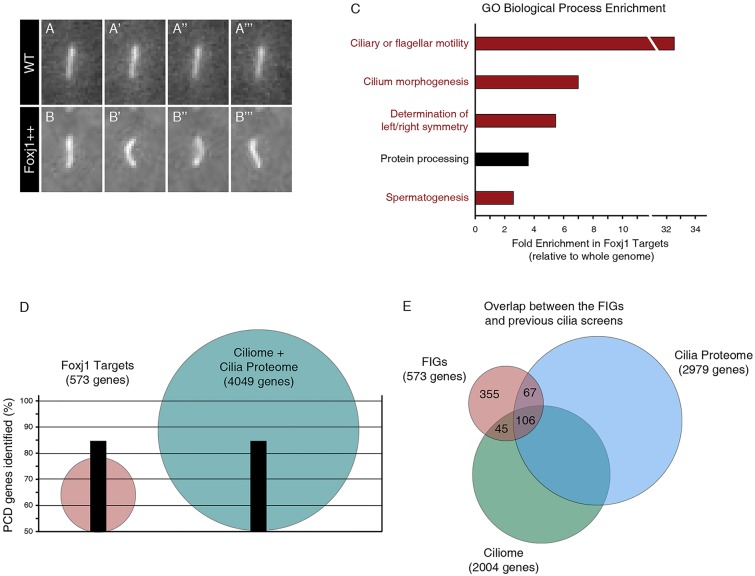
**Foxj1 upregulates known ciliogenic and ciliopathic genes.** (A-B‴) Sequential stills from movies of a *Tg(actinb2::Arl13b-GFP)* transgenic embryo showing (A-A‴) an immotile primary cilium on a wild-type muscle cell (WT) and (B-B‴) an ectopic motile cilium on a muscle cell of an *Tg(actinb2::Arl13b-GFP; hsp70::foxj1a)* embryo that overexpressed Foxj1 (Foxj1++). (C) GO annotation enrichment for the human orthologs of the Foxj1 target genes relative to the human genome. Of the top five enriched categories, four are directly related to cilia motility or morphogenesis (red). The other enriched GO category may be indirectly related to cilia (black). Categories are overlapping; however, redundant categories were not duplicated (see Materials and Methods). (D) The Foxj1 target genes are more efficient at identifying known PCD-causing genes than the ciliome and the cilia proteome combined. Although it contains fewer than 600 mammalian genes, the list of Foxj1 targets (red) contains 84.6% of PCD genes. By comparison, the ciliome and the cilia proteome together consist of 4049 genes (blue) and identify the same percentage of the known PCD genes as the Foxj1 targets. (E) There is little overlap between the FIGs (red) and the cilia proteome (blue) or the ciliome (green).

We generated transgenic zebrafish in which inducible Foxj1 misexpression led to the widespread production of ectopic motile cilia (Fig. 1A,B; supplementary material Movies 1 and 2). Foxj1 is thought to function principally as a transcriptional activator (Lim et al., 1997). Consistent with this, whole-transcriptome microarrays of embryos overexpressing Foxj1 revealed a striking upregulation of gene expression, with 662 genes significantly induced (supplementary material Fig. S1A). As our aim was to identify genes with potential relevance to ciliopathies, we removed Foxj1 targets without mammalian (mouse or human) orthologs, leaving 596 zebrafish genes, corresponding to 573 mammalian genes (supplementary material Table S1). We refer to this list, a combination of the direct and indirect targets of Foxj1, as Foxj1-induced genes (FIGs).

Using the gene ontology (GO) term ‘enrichment’, we found that four out of the top five over-represented classes of genes are directly linked to cilia, including ‘ciliary or flagellar motility’ and ‘cilium morphogenesis’ (GO:0001539 and GO:0060271; Fig. 1C). Expression analysis of the FIGs revealed that the number of genes expressed in multiple motile ciliated tissues is 2.6-fold enriched over the entire zebrafish genome (*P*=2.6×10^−9^, Fisher's Exact test; supplementary material Fig. S1B). We also manually annotated the list of FIGs, and found 83 known regulators of ciliary differentiation and function, with the human orthologs of 35 of these genes having been implicated in ciliopathies (supplementary material Table S2).

The results of 12 earlier major ciliary gene screens have been compiled into two databases: the ciliome (Inglis et al., 2006) and the cilia proteome (Gherman et al., 2006). When combined, these databases comprise over 4000 genes that contain 84.6% (22/26) of the known PCD genes. Strikingly, our collection of Foxj1 targets, consisting of just 573 genes, identifies the same percentage of the known PCD genes (Fig. 1D; supplementary material Fig. S1C), indicating that the FIGs have a high predictive capability for new PCD-causing genes, but with a lower false-positive rate. Additionally, the majority of the Foxj1 targets are putative novel cilia genes, with only 38.0% of the genes occurring in either of the existing ciliary databases (Fig. 1E). The combination of the ciliary GO term and tissue expression enrichment, and the predictive capability for known ciliary and PCD-causing genes suggests that the FIGs also contain novel motile cilia genes.

### Many FIGs are involved in various aspects of cilia formation and function

Among the FIGs are genes encoding proteins required for ciliary motility, including inner and outer dynein arm proteins and components of the nexin-dynein regulatory complex (N-DRC). In addition, there are numerous genes involved in ciliogenesis, such as genes encoding tubulin-modifying enzymes, components of the transition zone and intraflagellar transport (IFT) proteins (supplementary material Fig. S1D). The IFT genes represented in the FIGs include eight major subunits (*ift25/hspb11*, *ift27/rabl5*, *ift80*, *ift88*, *ift140*, *ift172*, *ift121/wdr35* and *traf3ip1*) and one IFT accessory gene (*ttc26*). As expected, protein domains found in ciliary proteins are among the most enriched domains in the FIGs, including the dynein heavy chain protein domain (IPR004273), the IQ calmodulin-binding region (IPR000048) and two domains that are found among IFT proteins: the TTC (Tetratricopeptide-like helical: IPR011990) and WDR (WD40 repeat: IPR001680) domains (supplementary material Fig. S2A).

When analyzing the entire set of FIGs, PANTHER protein characterization identified cytoskeletal proteins that could serve as structural components of cilia (78 genes), and possible regulators of ciliary differentiation and function, including transcriptional regulators (26 genes) and protein regulators such as kinases (19 genes), phosphatases (8 genes) and enzyme modulators (61 genes). Interestingly, nearly 12% of the FIGs consists of genes encoding predicted receptors and signaling molecules (67 genes), indicating that motile cilia may perform more sensory and signaling functions than previously recognized (Bloodgood, 2010) (supplementary material Fig. S2B).

In addition to these known and putative cilia genes, the FIGs also contain 44 genes without any identified structural or functional homology. Further analysis of these genes may open new pathways of study with respect to the molecular components and regulators of cilia.

### Selection of a random set of genes to functionally evaluate the entirety of the FIGs

Besides regulating motile cilia biogenesis, Foxj1 also has been suggested to have a non-ciliary transcriptional function in mammalian lymphocytes (Lin et al., 2004). In addition, the overexpression of transcriptional activators can cause the induction of non-physiological targets. In view of these possibilities, and because over 60% of the FIGs consist of genes not found in the ciliome or in the cilia proteome, we decided to undertake a detailed functional analysis of a large subset of this collection to fully establish their relevance to ciliary differentiation and function. We reasoned that selecting this subset of genes in an unbiased manner (using a computer-generated pseudo-random selection method; see Materials and Methods for details) would allow us to estimate accurately the ciliary roles of the entire collection of FIGs.

After removing the 83 known regulators of cilia function, we selected 53 of the remaining 513 genes at random and confirmed, using reverse transcription and quantitative PCR (RT-qPCR), that 50 of the 53 genes (94.3%) are upregulated by Foxj1 (*P*<0.05, Student's *t*-test; supplementary material Fig. S3 and Table S3). For simplicity, we refer to the FIGs by their zebrafish gene names. Where the gene name of the human or mouse ortholog is different from the zebrafish gene, the name of the ortholog is included in parentheses (supplementary material Table S3). During the preparation of this work, the designated mammalian ortholog of one gene, *BX470211.1*, was removed from the ENSEMBL database; however, we still include the analysis of this gene here. We carried out a full functional analysis of the randomly selected 50 genes, which consisted of examining the subcellular localization of the encoded proteins and determining the phenotypes of zebrafish embryos deficient for the genes.

### Proteins encoded by a subset of the FIGs localize to the ciliary apparatus

**Fig. 2.**
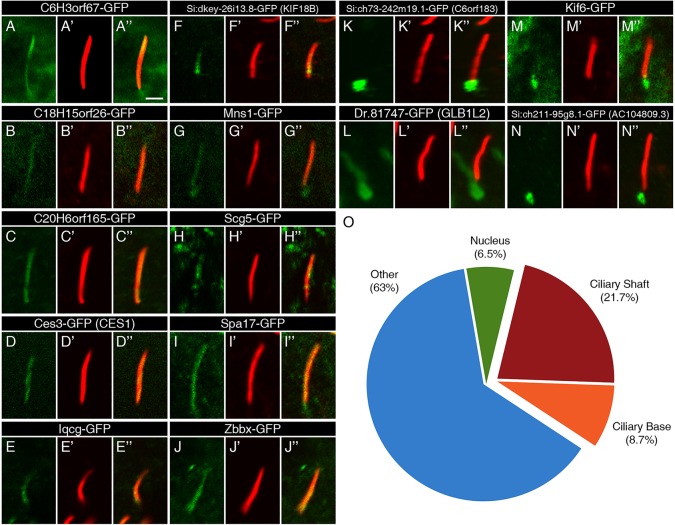
**Ciliary localization of FIG-encoded proteins in the zebrafish embryo.** (A-N) The localization of GFP-tagged FIG-encoded proteins relative to motile cilia of the KV (green) in 13-14 hpf embryos. The GFP signal was amplified with anti-GFP antibodies. (A′-N′) Ciliary axonemes were labeled with anti-acetylated tubulin antibodies (red). (A″-N″) Overlays of the two channels show the overlap between the gene products and the ciliary shafts. Scale bar: 2 µm. (O) An overview of the localization of the FIG gene products shows that the majority does not localize to the cilium. These genes may encode cytoplasmic, membrane or nuclear regulators of cilia function.

We injected *in vitro*-synthesized mRNAs encoding each of the 50 genes with a GFP tag into zebrafish embryos and assayed localization by immunofluorescence in motile cilia of Kupffer's vesicle (KV), the teleost equivalent of the mammalian node (supplementary material Table S3; see Materials and Methods). The localization patterns of 14 proteins either overlapped with, or were associated with, cilia (Fig. 2). Ten proteins localized along the extracellular shafts of the motile cilia (Fig. 2A-J). Four additional proteins localized to the base of the cilia, in close proximity to the basal body (Fig. 2K-N). Surprisingly, the remaining proteins showed no association with ciliary structures: three were predominantly nuclear (supplementary material Fig. S4A-C), whereas the remainder appeared to localize to a combination of the cytoplasm, nucleus and cell membrane (supplementary material Fig. S4D-FF and Table S3) when compared with uninjected embryos (supplementary material Fig. S4GG). To establish expression levels, seven proteins that yielded low immunofluorescence signals were assessed by western blot analysis. Three of these proteins showed significant expression, whereas four proteins were removed from the analysis due to undetectable expression levels (supplementary material Fig. S5).

Overall, we found that 30.4% of the proteins encoded by the FIGs localize to cilia. Extrapolation of these data to the remaining FIGs indicates that 30.4%±12.7% (95% confidence interval) of the proteins encoded by the FIGs localize to cilia. Therefore, it is likely that most of the FIGs encode novel membrane, cytoplasmic or nuclear regulators of cilia formation and function. This view is corroborated by recent discoveries of several PCD genes that encode cytoplasmically localized proteins that function in the preassembly of axonemal dyneins (Mitchison et al., 2012; Moore et al., 2013; Omran et al., 2008; Zariwala et al., 2013). The observed 30.4% ciliary localization is likely to be an underestimation, as steric interference from the C-terminal GFP tag may affect the endogenous pattern of protein localization. In addition, we have screened for localization only in the motile cilia of KV, and ciliary proteins have been shown to localize differentially in cilia of distinct cell types.

### Inactivation of FIGs suggests unique molecular signatures of motile cilia

**Fig. 3.**
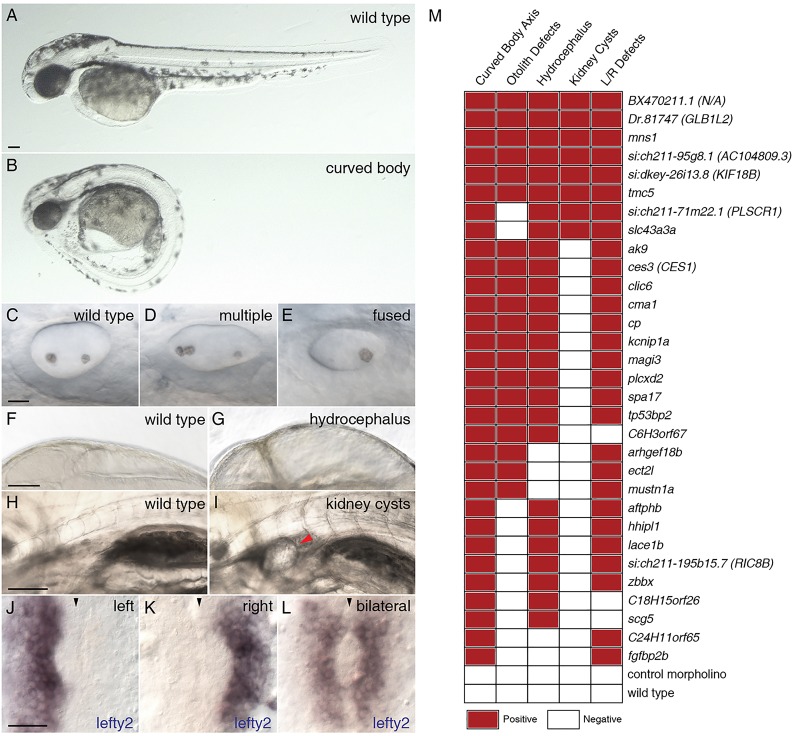
**Morpholino knockdown of FIGs causes multiple ciliary dysfunction-associated phenotypes in zebrafish embryos.** Five ciliary phenotypes were scored in the zebrafish embryos injected with morpholinos targeting the 50 selected FIGs. The extent of body axis curvature: (A) wild type or (B) curved body. Otolith defects in the inner ear: (C) two otoliths, (D) greater than two otholiths or (E) a single otolith. Swelling of the brain ventricles (hydrocephalus): (F) normal or (G) hydrocephalus. Kidney cysts: (H) no cysts or (I) a minimum of one cyst per embryo. Expression of *lefty2*: (J) leftward expression, (K) rightward expression or (L) bilateral expression or no expression (data not shown). The red arrowhead indicates the position of a kidney cyst (I); the black arrowheads indicate the midline (J-L). Scale bars: 100 µm in A,F,H; 20 µm in C,J. (M) Morpholino knockdowns of 31 genes exhibited defects in at least two of the assayed phenotypes. For morphological phenotypes, each square represents at least 60 assayed embryos from two independent injections. For left-right asymmetry defects, each square represents a minimum of 30 stained and scored embryos. A red square indicates that the morphant phenotype level was significantly different from the wild type and met a minimum defective percentage (see Materials and Methods).

We tested the role of the 50 randomly selected FIGs in cilia formation and function by injecting antisense morpholinos into zebrafish embryos (supplementary material Table S3). After several rounds of morpholino design and testing, we established that morpholinos directed against 48 of the genes reliably induced aberrant splicing, indicative of efficient inhibition of gene function (supplementary material Fig. S6). Translation-blocking morpholinos were designed to target the remaining two genes. Carrying out a functional phenotypic screen in zebrafish embryos allowed us to assay for an array of developmental and physiological defects caused by motile cilia abnormalities. These include curving of the body axis (Fig. 3A,B), perturbation of otolith formation in the inner ear (Fig. 3C-E), hydrocephalus (Fig. 3F,G), formation of kidney cysts (Fig. 3H,I) and improper establishment of left-right asymmetry (Fig. 3J-L) (Kramer-Zucker et al., 2005).

Strikingly, we found that the knockdown of the majority of the genes (62%) showed at least two motile cilia dysfunction-associated phenotypes (Fig. 3M). Nearly 20% of the genes exhibited all five of the phenotypes and are likely to be core motile cilia genes. Interestingly, the remaining genes displayed subsets of motile cilia phenotypes upon knockdown, allowing us to parse the molecular signatures of different types of motile cilia. For example, novel genes such as *si:ch211-71m22.1* (*PLSCR1* in human) and *slc43a3a* are required for the function of all motile cilia, except for those in the otic vesicle, whereas two genes encoding putative guanine nucleotide exchange factors, *arhgef18b* and *ect2l*, are needed for the function of motile cilia in just the developing ear and KV.

During the preparation of this work, four of the randomly selected FIGs were shown to be required for ciliogenesis in the mouse, *Xenopus* or zebrafish: *Mns1* (Zhou et al., 2012), *IQCG* (Harris et al., 2014), *C18H15orf26* (Austin-Tse et al., 2013) and *ccdc78* (Klos Dehring et al., 2013). These studies have provided independent validation that our collection of FIGs is enriched for novel ciliary genes. Conversely, our functional screen has confirmed that, in addition to being involved in cilia formation in the mouse, at least two of these genes – *Mns1* and *Iqcg* – are also required for motile cilia function in the zebrafish.

Based on the inactivation of the random subset of the FIGs, we estimate that 62%±12.8% of the population of 513 novel genes (the FIGs with the 83 known cilia genes removed) has a ciliary function (95% confidence interval). From this, we can conclude that the FIGs consist of between 252 and 383 new genes with a role in cilia formation or function.

### Function of FIGs in motile cilia formation and motility

**Fig. 4.**
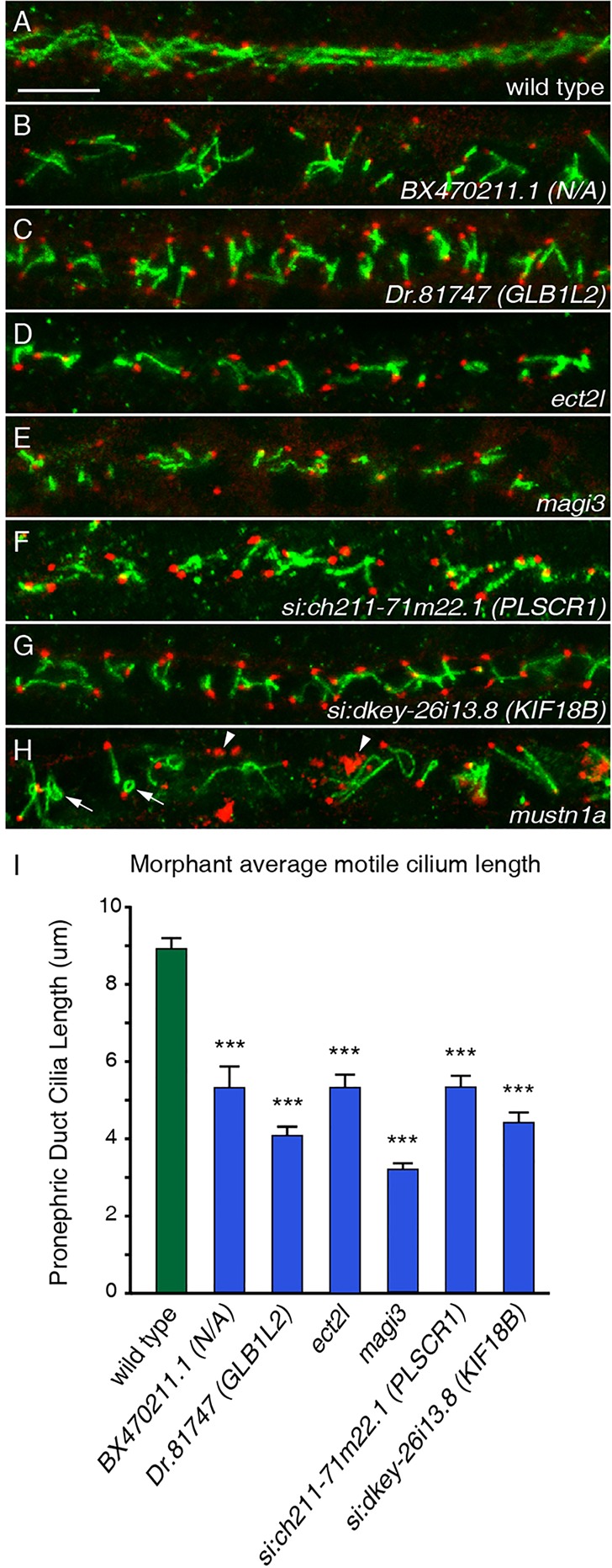
**FIGs are required for both ciliogenesis and cilia organization.** (A-H) Cilia of the pronephric duct were stained with anti-Arl13b antibodies (green) and the basal bodies were stained with antibodies to γ-tubulin (red) in 24 hpf embryos. (A) Long cilia of uniform length and orientation are visible in a wild-type embryo. (B-G) Six morphants exhibited shortening of pronephric cilia at 24 hpf. (H) The knockdown of *mustn1a* caused curling of cilia (arrows) and a disorganization of the γ-tubulin (arrowheads). Scale bar: 10 µm. (I) Measurements of ciliary length in wild-type versus morphant embryos for six genes reveals the extent of ciliary shortening in the morphants. Error bars represent s.e.m. ***P<5.0×10^−7^ (Student's *t*-test, two-tailed, *P*-values are listed in supplementary material Table S3); *n*≥30.

In order to explain the observed developmental and physiological phenotypes linked to ciliary dysfunction, we screened for abnormalities in morphology of the ciliary shaft and basal bodies in morphant embryos by immunofluorescence microscopy. We found that the loss of six genes – *BX470211.1*, *Dr.81747* (*GLB1L2* in human), *ect2l*, *magi3*, *si:ch211-71m22.1* and *si:dkey-26i13.8* (*KIF18B* in human) – showed a significant (*P*<5.0×10^−7^, Student's *t*-test, two-tailed) reduction in the length of motile cilia in the kidney tubules (Fig. 4A-G,I; supplementary material Table S3). In addition, embryos deficient for *mustn1a,* which encodes a transcription factor, showed curled cilia and disorganized γ-tubulin expression, a marker of the basal bodies (Fig. 4H).

**Fig. 5.**
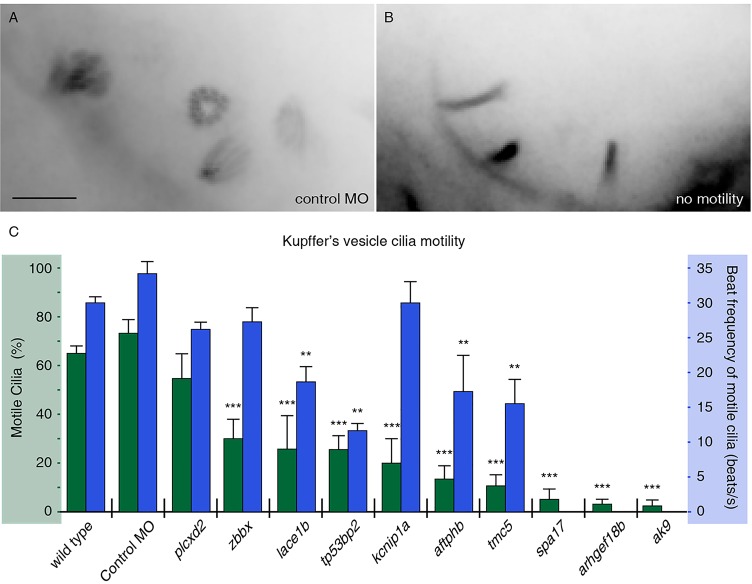
**FIGs are required for ciliary motility.** (A,B) Embryos were injected with RNA encoding Arl13b-GFP to label KV cilia and imaged using high framerate video microscopy. (A) An overlay of 20 frames from a movie from a control embryo indicates four moving cilia in view. (B) By comparison, overlays from movies of an embryo with immotile cilia show no blurring, indicating stationary objects. Scale bar: 5 µm. (C) Measurements of ciliary motility in wild-type and morphant embryos targeting ten selected genes. Two aspects of ciliary motility were scored: the percentage of KV cilia that exhibited motility (green) and the average beat frequency of motile cilia (blue). Error bars represent s.e.m. ***P<4.0×10^−7^ (Fisher's Exact test, two-tailed; *P*-values are listed in supplementary material Table S3). **P<5.0×10^−3^ (Student's *t*-test, two-tailed, *P*-values are listed in supplementary material Table S3). The three genes without reported ciliary beat frequency did not have sufficient motile cilia for analysis (*n*<8).

We hypothesized that the remainder of the genes affects ciliary motility. To test this possibility, we assayed motility by fluorescently labeling cilia in live embryos and then performing high-frame rate video microscopy. We selected the ten morpholinos that exhibited the most severe left-right asymmetry abnormalities (excluding the genes that gave ciliary shortening phenotypes) for examining ciliary motility in KV. We co-injected the morpholino and mRNA encoding Arl13b-GFP into the embryos to label the cilia of morphant embryos. Motile cilia of KV were imaged at 14-15 hpf at speeds up to 500 frames per second, and analyzed for two aspects of motility: percentage of KV cilia that exhibit motility, and the average beat frequency of these cilia. Wild-type and control morpholino-injected embryos showed ∼60-70% of KV cilia motility at 14-15 hpf, with beat frequencies averaging around 30 Hz (Fig. 5A,C; supplementary material Table S3). The observed beat frequencies correspond to previously published reports (Kramer-Zucker et al., 2005). However, upon morpholino-mediated knockdown of nine out of the ten selected genes, the percentage of motile cilia decreased to as low as 2% (Fig. 5B,C; supplementary material Movies 3-14 and Table S3). In addition, cilia beat frequencies were significantly lower in four of the morphants when compared with wild-type embryos (Fig. 5C; supplementary material Table S3).

Together, these data indicate that many of the FIGs affect either cilia length or ciliary motility. Indeed, of the 31 genes that produce phenotypes linked to dysfunction of the motile cilia upon knockdown, we have demonstrated that at least 16 of these genes affect the length, beating or organization of motile cilia.

**Fig. 6.**
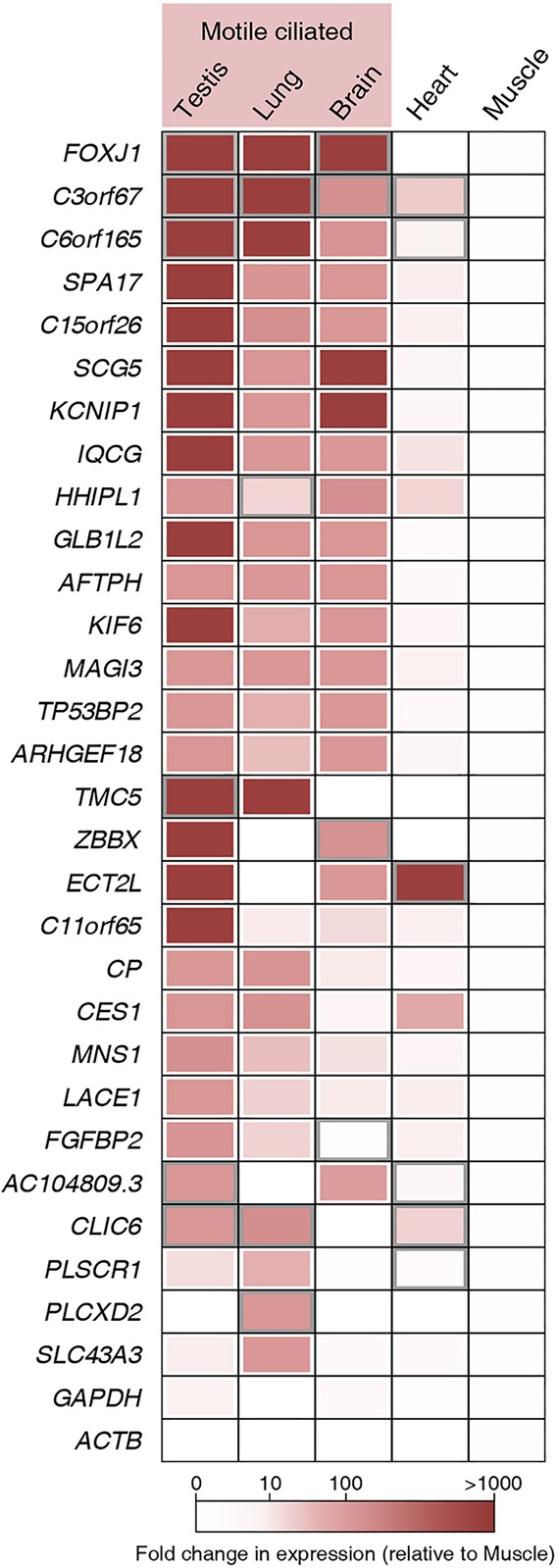
**FIG expression is enriched in human tissues that have motile cilia.** Expression levels of 28 genes (whose knockdown in zebrafish produced ciliary phenotypes and/or whose protein products localized to cilia) were assayed using qPCR on human cDNA libraries from various human tissues. The expression of each gene was normalized to *ACTB* levels, and then measured relative to expression levels in skeletal muscle tissue. Five tissues were assayed, three with motile cilia (testis, lung and brain) and two with no motile cilia (heart and skeletal muscle). Fold change for each tissue relative to muscle is given as a color, in a range from more than 1000-fold enriched (dark red) to no significant enrichment or no significant data (white). Boxes outlined in white have a significance of *P*<0.05; boxes outlined in gray have a significance level of 0.05≤*P*<0.09 (Student's *t*-test, two-tailed; *P*-values are listed in supplementary material Table S3); each square represents three to six technical replicates. *FOXJ1* was included as a positive control; *GAPDH* was included as a negative control.

Since the expression of genes that have roles in motile cilia formation and function tend to be enriched in tissues with motile cilia, we assayed the expression of the human orthologs of 28 genes that produced ciliary phenotypes or encoded cilia-localized proteins in zebrafish embryos. We found that 26 (92.9%) of these genes are induced in at least two human motile ciliated tissues (Fig. 6; supplementary material Table S3). These genes are likely to be involved in motile cilia functions in humans, making them excellent novel candidate genes for PCD.

## DISCUSSION

By analyzing the genes transcriptionally upregulated by Foxj1, the master regulator of the motile ciliogenic program, we have discovered a collection of genes that we have termed the FIGs. Several core metrics demonstrate the uniqueness and value of the FIGs in furthering our understanding of ciliary biology and the etiology of ciliopathies.

The first distinctiveness of the FIGs lies in the extensive coverage, relative to previous cilia screens, of genes that are known to be mutated in PCD. This feature emerges from the fact that the FIGs represent the core network of genes involved in vertebrate motile cilia formation and function, connected through the activity of the central transcriptional regulator Foxj1 (Choksi et al., 2014). Next, the minimal overlap between the FIGs and the existing ciliary gene databases indicates that the majority of the FIGs consists of novel ciliary genes. Many of the earlier cilia gene screens relied on proteomics of cilia or basal bodies, whereas the screen presented here detects structural components, as well as cytoplasmic and nuclear regulators, of ciliogenesis and ciliary function. Of the other ciliome and cilia proteome screens, those based on informatics and transcriptional approaches were restricted to genes present in the flagellated green alga *Chlamydomonas* (a protozoan) or the nematode *C. elegans*, which lacks motile cilia and motile cilia genes. However, the FIGs comprise genes present in the genomes of vertebrates. More than 60% of the FIGs are not present in *Chlamydomonas* (supplementary material Table S1), which does not use a Foxj1-dependent transcriptional program for flagellar assembly (Vij et al., 2012).

Besides the ciliome and the cilia proteome databases, studies over the recent years have identified genes expressed in the motile multiciliated respiratory epithelial cells of mice (Hoh et al., 2012), genes upregulated by the multiciliated cell fate determinant Multicilin in the *Xenopus* epidermis (Stubbs et al., 2012), and genes regulated by Foxj1 in the *Xenopus* epidermis (Stubbs et al., 2008) or the developing mouse brain (Jacquet et al., 2009). In addition, the target genes of another ciliogenic transcription factor, Rfx2, which is upregulated by Foxj1 (supplementary material Table S1) (Yu et al., 2008), have been analyzed in the context of the multiciliated cells of *Xenopus* (Chung et al., 2014). The FIGs remain distinct from each of these datasets, with a maximum of 22.9% of the FIGs represented in any of these previous reports (supplementary material Fig. S7A-D) and a total of 38.4% of the FIGs represented in all of these datasets combined. In addition, the FIGs identify a greater proportion of the known PCD genes than any of these previous screens (supplementary material Fig. S7E).

**Fig. 7.**
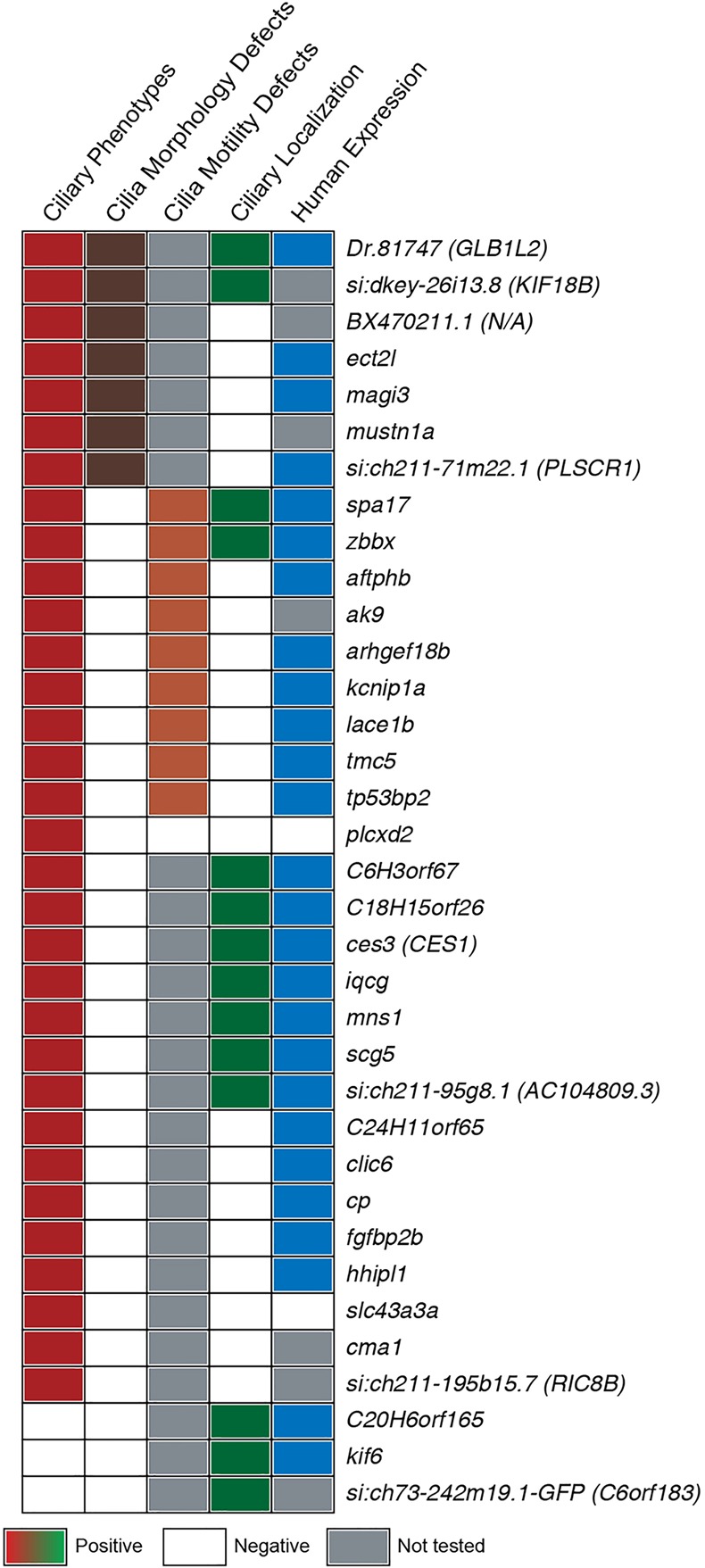
**A summary of the functions of the randomly selected FIGs.** An overview of the functional analysis of the selected FIGs is given. The 35 genes that produced ciliary phenotypes upon knockdown or encoded proteins with cilia-associated localization patterns are listed. Ciliary phenotypes indicates genes that, when knocked down in the zebrafish, produce at least two motile cilia-related phenotypes (red). Cilia morphology defects indicates genes that, when knocked down, show cilia length or organization defects in the pronephric duct (brown). Cilia motility defects indicates genes that, when knocked down, show reduced KV cilia motility (orange). Ciliary localization indicates genes whose encoded proteins show cilia-associated localization upon overexpression (green). Finally, human expression indicates genes whose human ortholog expression is enriched in at least two motile ciliated tissues relative to a non-motile ciliated tissue (blue).

Finally, in the absence of in-depth functional validation, it is unclear how many of the genes identified in any of the previous screens are genuinely linked to cilia. By contrast, we have functionally validated the FIGs extensively in a vertebrate organism (summarized in Fig. 7). In a detailed analysis undertaken on a random selection of 50 novel genes, we found 62% produced ciliary phenotypes in zebrafish embryos when inactivated. The fact that our selection was unbiased means that we can extrapolate to the entire set of FIGs and affirm that we have found roughly 300 new ciliary genes. Together, these arguments reinforce the idea that the FIGs consist of the most complete vertebrate motile cilia gene network assembled to date.

The identification of genes regulating the formation of functional vertebrate motile cilia has been problematic due to the structural and functional complexity of the motile cilium and difficulties in the *in vitro* culture of motile ciliated cells from different tissues. However, by leveraging on the power of the master regulator of motile ciliogenesis, Foxj1, our targeted screen in an intact vertebrate model has yielded a novel, comprehensive and functionally validated collection of ciliary genes that will both further our understanding of cilia structure and function, and aid the discovery of novel ciliopathy genes.

## MATERIALS AND METHODS

### Zebrafish husbandry

*Danio rerio* of the AB strain were reared at 28°C unless otherwise mentioned. Embryos were obtained from natural matings and cultured in egg water with or without 1-phenyl-2-thiourea (PTU; Sigma, #P-7629) to prevent pigmentation. Adult zebrafish used in this study were between 3 and 9 months old. All zebrafish experiments were approved by the Singapore National Advisory Committee on Laboratory Animal Research.

### Transgenic zebrafish for Foxj1 target screen

The zebrafish *foxj1a* cDNA (zebrafish have two paralogous Foxj1 genes, *foxj1a* and *foxj1b*) was cloned downstream of the heat shock promoter (*hsp70*) followed by an IRES GFP to serve as a reporter. This construct was injected into freshly fertilized zebrafish eggs with the I*-Sce*I meganuclease to facilitate transgenesis as described previously (Thermes et al., 2002). *Tg(hsp70::foxj1a)* F2 embryos were subject to two consecutive heat shocks at 37°C for 1 h each at 18 and 20 h post-fertilization (hpf). GFP-positive embryos were selected using a fluorescent stereomicroscope for microarray or qPCR analysis.

### Microarray sample preparation and analysis

Fifty wild-type or *Tg(hsp70::foxj1a)* embryos were dechorionated at 22 hpf and RNA was extracted using the RNeasy Mini Kit (Qiagen, #74106). Total RNA (10 µg) was used to make double-stranded cDNA using the Superscript III Double-Stranded cDNA Synthesis Kit (Invitrogen, #11917-020). The cDNA was labeled with the Nimblegen Labeling Kit and hybridized to Nimblegen Zebrafish whole-genome 60-mer oligonucleotide 12×135k arrays (Roche, #071105). Hybridization was carried out overnight at 42°C in a Maui Hybridization System (BioMicro Systems) and arrays were scanned with a GenePix 4000B scanner (Molecular Devices), with a pixel size of 5 µm.

Microarray analysis was performed using the ArrayStar software (DNAStar). RMA analysis was used to normalize the array, and six biological replicates were averaged for each sample. Probes greater than twofold upregulated in the *Tg(hsp70::foxj1a)* sample with a *P*-value of <0.01 were deemed to represent Foxj1 target genes (Student's *t*-test, corrected for Multiple Hypothesis Testing using Benjamini Hochberg False Discovery Rate correction). The microarray data are available at Array Express under accession number E-MTAB-2815. Microarray data for Foxj1 target genes are provided in supplementary material Table S1.

### Microarray annotation and gene analysis

Probes were mapped against version 9 of the zebrafish genome using BLAST. Probes with two or fewer mismatches to the database sequence were accepted. Ensembl gene models were used in most instances; if unavailable, Unigene gene models and IDs were used instead. Orthology was determined using Ensembl human or mouse ortholog or predicted ortholog. When two or more orthologs were predicted, only the vertebrate gene with the highest level of identity was used. For genes without an Ensembl ortholog, Unigene, OrthoDB or InParanoid-predicted orthologs were used.

GO analysis was performed on the human orthologs of the Foxj1 targets, with a background of the entire human genome using DAVID (http://david.abcc.ncifcrf.gov/). The GO Biological Process was investigated and GO categories with a minimum of five genes, at least a 2.5-fold enrichment, and *P*≤0.02 were reported (Fisher's Exact test). Any redundant categories, defined as a category with more than 80% overlap with another category, were removed for clarity. Enriched classes of Interpro protein domains with a minimum of 10 genes, at least a twofold enrichment, and with a *P*≤0.01 were reported (Fisher's Exact test). Redundant categories were removed for clarity. Protein classes were defined using the PANTHER database (http://www.pantherdb.org/).

### Random gene selection for functional analyses

In order to select the random 53 genes for analysis, we first removed the genes that had been previously linked to cilia from the list of Foxj1-induced genes, leaving 513 genes. We then sorted the 513 genes by their unique Nimblegen microarray ID number (SEQID). Using the RAND function (a pseudorandom number generator) in MS Excel 2007, we generated a list of 513 unique numbers, with a single number assigned to each SEQID number. Finally, we sorted the genes by their unique random number, and selected the first 53 genes (a number established prior to the ordering of the list) for qPCR and further functional analysis.

### Quantitative PCR (qPCR)

For zebrafish qPCR, embryos were collected under the same conditions as for the microarray analysis. Total RNA (2 µg) was used for cDNA synthesis by Superscript III (Invitrogen, #18080-051). Quantitative PCR was performed on a Fast 7900HT real-time machine (Applied Biosystems) using a Fast SYBR green master mix (Applied Biosystems, #4385612). Primers for qPCR were designed to flank exon-intron junctions to also test for genomic DNA contamination (supplementary material Table S3). Three to six biological replicates were measured for each gene.

For human qPCR analysis, commercially available human cDNA libraries were used (Origene). Primers for qPCR were designed to flank exon-intron junctions to test for genomic DNA contamination (supplementary material Table S3). Three to six technical replicates were measured for each gene. Significance was determined using a Student's *t*-test, with *P*<0.05 deemed highly significant, and 0.05≤*P*<0.09 deemed significant.

### RACE/EST

Rapid amplification of cDNA ends (RACE) was conducted on cDNA libraries made from 18-20 hpf embryos using the FirstChoice RLM-RACE kit (Ambion, #AM1700M). PCR bands were cloned using the TOPO-TA cloning system (Invitrogen, #450641) and sequenced. cDNA sequences were assembled using a combination of Ensembl gene predictions and EMBL ESTs as references.

### *In situ* hybridization

The *lefty2* RNA *in situ* probe was made by cloning a fragment of the cDNA. The *lefty2* plasmid was cut with *Bam*HI and antisense, DIG-labeled RNA was transcribed using the T7 RNA polymerase (Roche, #10881767001) and DIG RNA labeling mix (Roche, #11277073910).

Wild-type or morpholino-injected embryos were fixed at 24 hpf in 4% PFA (in PBS) overnight at 4°C. Embryos were digested in proteinase K then fixed again in 4% PFA. DIG-labeled *in situ* probes were hybridized in a formamide-based hybridization buffer overnight at 65°C. DIG probes were detected using an anti-DIG antibody coupled to alkaline phosphatase (Roche, #11093274910). *In situ* hybridization experiments were developed using a colorimetric assay and scored under a stereomicroscope.

A minimum of 30 embryos were scored for each batch of morpholino-injected embryos. After tallying the *lefty2* expression patterns, morphants were checked for significant difference from wild-type embryos with a Fisher's Exact test (2×2 matrix, two-tailed). For left-right asymmetry defects, positives were morphants with more than 20% of the embryos exhibiting aberrant *lefty2* expression, with *P*<2.0×10^−3^.

### Morpholino design, injection and testing

Morpholinos were designed and synthesized by GeneTools (supplementary material Table S3). Splice-blocking morpholinos were designed for most genes, except for two where translation-blocking morpholinos were designed because splice-blocking morpholinos were not possible. Morpholinos were tested by injecting varying amounts into freshly fertilized zebrafish eggs and determining the maximum sublethal dose (supplementary material Table S3). RNA was then extracted and converted to cDNA. Primers were designed surrounding the morpholino binding site for PCR amplification (supplementary material Table S3). PCR was optimized for each primer pair, and amplified bands were sequenced to confirm deletions or insertions.

### Morphological phenotype analysis

Phenotypes were scored in live wild-type or morpholino-injected embryos using a dissection stereomicroscope. Phenotypes were analyzed in embryos of different stages. Otolith counts were performed at 20-22 hpf. Curved body axis and hydrocephalus were determined at 48 hpf. Kidney cysts were scored 4-5 days post-fertilization (dpf). For each phenotype, a minimum of two independent injections were scored, with at least 30 embryos for each trial. Morpholino injection trials with greater than 25% lethality at 24 hpf were discarded, and the morpholino dose was adjusted to reduce lethality. Injections were coded and randomized; however, the morphological effects of morpholino injection were too evident for blind phenotype scoring. To evaluate significance, we used the Fisher's Exact test (2×2 matrix, two-tailed) to compare the injected embryos with wild-type embryos. Phenotype designations were made with a combination of a minimum percentage of defective embryos and a maximum *P*-value when the morphant embryos were compared with wild-type embryos. For curved body phenotypes, positives were morphants with more than 15% aberrant embryos, with *P*<3.0×10^−10^. For otolith phenotypes, positives were morphants with more than 20% aberrant embryos, with *P*<4.0×10^−7^. For hydrocephalus phenotypes, positives were morphants with more than 10% aberrant embryos, with *P*<3.0×10^−11^. For kidney cyst phenotypes, positives were morphants with more than 10% aberrant embryos, with *P*<6.0×10^−5^. Specific *P*-values and phenotype counts for each morpholino are listed in supplementary material Table S3.

### mRNA transcription and injection

cDNAs were synthesized (Sigma) and cloned upstream of a GFP tag; they were then subcloned into a vector for transcription, either pSP6-4T or pCS2+. Plasmids were linearized and then transcribed and capped with the mMessage SP6 kit (Ambion, #AM1340). mRNAs were purified and resuspended in nuclease-free water and 50-400 pg of the RNA was injected into each fertilized zebrafish egg.

### Immunohistochemistry

Embryos were fixed for 2 h at room temperature in 4% paraformaldehyde in PBS (PFA). Embryos were stored in methanol, and then rehydrated in a gradient of PBS/methanol. Embryos were permeabilized in acetone, blocked in PBDT (PBS, 1% BSA, 1% DMSO and 1% Triton X-100) plus 5% sheep serum. Primary and secondary antibodies were incubated and washed in PBDT.

Primary antibodies used include mouse anti-acetylated-α-tubulin 6-11B-1 (1:500 for zebrafish embryos, Sigma, #T6793), mouse anti-γ-tubulin GTU-88 (1:500, Sigma, #T6557), rabbit anti-GFP (1:500, Abcam, #ab6556; Torrey Pines Biolabs, #TP401), rabbit anti-Arl13b (Duldulao et al., 2009) (1:200), DAPI (4′,6-diamidino-2-phenylindole; Molecular Probes, #D1306) was used to label cell nuclei. Alexa fluorophore-labeled anti-rabbit or anti-mouse secondary antibodies (Molecular Probes) were used to visualize staining.

### Protein extraction and western blot

Embryos overexpressing a FIG-encoded protein tagged with GFP were manually dechorionated at 13-14 hpf. Embryos were washed twice in PBS then homogenized in SDS loading buffer. Proteins were denatured at 95°C for 10 minutes before flash freezing. Protein extracts were probed with the following antibodies: rabbit anti-GFP (1:3000, Abcam, #ab6556) and mouse anti-Actin AC-40 (1:3000, Sigma, #A4700).

### Imaging

Immunofluorescence images were captured on an Olympus Fluoview 1000 or a Zeiss Apotome Axiovert Z1 microscope with 60×, 1.4 NA oil immersion objectives. Bright-field images were captured with a Zeiss Axioplan 2 compound microscope using a Nikon DMX1200 digital camera. Live imaging of cilia were performed in *Tg(actinb2::Arl13b-GFP)* embryos or in embryos injected with 188 pg of RNA encoding Arl13b-GFP (Borovina et al., 2010) using a Zeiss Axioplan 2 compound microscope fitted with either an Evolve 512 EMCCD camera (Photometrics) or an Orca Flash 4.0 sCMOS camera (Hamamatsu). Images were processed in Adobe Photoshop. Cilia length measurements and movie editing were performed using ImageJ. Figures were assembled using Adobe Illustrator.

### Statistical analyses

Statistical tests used for each experiment are listed in the appropriate section of the Materials and Methods. Briefly, independent *t*-tests were used to test differences in continuous, normally distributed data: microarray expression levels, qPCR expression levels, cilia length measurements and cilia beat frequencies. Multiple hypothesis testing correction was performed in the case of the microarray analysis. Fisher's Exact test using 2×2 contingency tables was used to compare categorical and independent data: GO analysis, gene expression enrichment, protein domain enrichment, morphant phenotype scoring and ciliary motility.

## Supplementary Material

Supplementary Material
