# Supplementary material for: Systematic discovery of novel ciliary genes through functional genomics in the zebrafish
Source: Development. 2014 Sep;141(17):3410–9. doi: 10.1242/dev.108209 (PMC4199137; doi:10.1242/dev.108209)
Supplement: Supplementary Material [file supp_141_17_3410__index.html]

Systematic discovery of novel ciliary genes through functional genomics in the zebrafish — Supplementary Material 

# Systematic discovery of novel ciliary genes through functional genomics in the zebrafish

## DEV108209 Supplementary Material

**Files in this Data Supplement:**

- **Supplementary Material**
