# Supplementary material for: Systematic discovery of novel ciliary genes through functional genomics in the zebrafish
Source: Development. 2014 Sep;141(17):3410–9. doi: 10.1242/dev.108209 (PMC4199137; doi:10.1242/dev.108209)
Supplement: Supplementary Material [file supp_141.17.3410_DEV108209.pdf]

Supplementary Legends

Supplementary Fig. S1. An overview of the FIGs. (A) Expression levels of each gene on the microarray visualized by scatter plot to evaluate trends in the data. Each spot on the microarray is represented by a single point on the graph. The expression levels of genes in the Foxj1 overexpression embryos are plotted on the y-axis while wild type expression levels are on the x-axis. Expression levels are given in arbitrary units. Red dots represent genes upregulated in Foxj1 overexpression embryos, yellow dots represent genes expressed at similar levels between Foxj1 overexpression and wild type embryos, while blue dots represent genes downregulated in Foxj1 overexpression embryos. (B) Many of the Foxj1-induced genes are expressed in multiple ciliated tissues of the developing zebrafish embryo. By searching the zebrafish gene expression database (available at http://zfin.org/), we find that 32.8% of the FIGs found in the expression database are expressed in at least two ciliated tissues (61 out of 186). In comparison, only 12.4% of all genes catalogued in the database are expressed in at least two ciliated tissues (1,368 out of 11,036). This represents a 2.6-fold enrichment of genes with expression patterns in ciliated tissues within the FIGs ($p = 2.6 \times 10^{-9}$, Fisher's Exact Test). (C) Twenty eight loci have been associated with PCD in the Online Mendelian Inheritance in Man (OMIM) database. Causative mutations in 26 genes have been mapped, and 22 of the genes figure in the Foxj1 target list (green), while just four are absent (grey). (D) The FIGs include numerous known regulators of ciliogenesis and cilia function. Eight

major classes of ciliary genes are listed, along with the genes upregulated by Foxj1 in each category.

Supplementary Fig. S2. Unbiased classification of FIG protein domains and proteins. (A) To further characterize the FIGs, we used Interpro to identify eight major protein domains which are enriched within the human orthologs of the FIGs compared to the human genome. Protein domains were at least 2-fold enriched in the FIGs compared to the human genome with $p < 0.01$. (B) Protein Class analysis of proteins encoded by FIGs using PANTHER (http://www.pantherdb.org/) reveals enriched classes of proteins in the human orthologs of the FIGs. The major categories of proteins are depicted, along with their Panther classification number and the number of genes present in each category.

Supplementary Fig. S3. FIGs are enriched in embryos overexpressing Foxj1 as measured by microarray analysis and RT-qPCR. Fifty three genes were selected randomly from the set of genes up-regulated by Foxj1 as determined by microarray analysis. The induction of 50 of these genes was confirmed using reverse transcription and quantitative (RT-qPCR) to compare transcript levels in embryos overexpressing Foxj1 to wild type embryos. These 50 genes showed a substantial increase in expression in response to Foxj1 overexpression. Blue bars indicate the expression fold change as measured by microarray analysis while red bars indicate expression changes measured by RT-qPCR. The y-axis is in a logarithmic scale

($\log_{10}$), error bars are standard errors of the mean. All results are significant by a Student's t-test ($p < 0.05$).

Supplementary Fig. S4. Localization of non-ciliary FIG-encoded proteins in zebrafish embryos. (A-GG) The localization of GFP-tagged FIG-encoded proteins in cells surrounding KV of 13-14 hpf embryos. The GFP signal was amplified with anti-GFP antibodies (green). (GG) Uninjected wild type embryos were stained and imaged under comparable conditions as controls. (A'-GG') Nuclei were labelled with DAPI (blue) and the overlay of the two channels show the overlap between the gene products and the nuclei, if any exists. Images are grouped by similar localization patterns. The scale bar in A' represents 10 μm.

Supplementary Fig. S5. Expression level of a subset of FIG-encoded proteins. Western blot analysis was used to detect protein expression levels for seven of the FIGs which showed low levels of expression by immunofluorescence. Three of these proteins showed a significant band when embryo extracts were probed with an antibody recognizing GFP. Wild type and GFP-positive extracts are included as negative and positive controls, respectively. Red asterisks mark bands that are near the expected protein size. The position and size of the protein standards are indicated. An antibody recognizing Actin was used as a loading control (LC).

Supplementary Fig. S6. Morpholinos against FIGs induce aberrant splicing or degradation of the targeted mRNAs in zebrafish embryos. Splice morpholinos were designed against 48 of the 50 randomly selected FIGs. Each morpholino was injected into zebrafish embryos and RNA was extracted at 24 hpf in order to verify that the morpholino is able to cause mis-splicing. PCR was used to check the resultant cDNA near the predicted morpholino binding site. Comparing the wild type (wt) amplification to the morphant (mo), we found that all of the morpholinos are able to induce either a substantial amount of mis-splicing or the degradation of the endogenous transcript. Reactions without reverse transcriptase are included (no RT) to eliminate the possibility of genomic DNA contamination, while *actb1* primers were used as loading controls (LC) to ensure equal amounts of starting material. Bands marked with a red asterisk were cloned and sequenced in order to verify the inclusion/exclusion of intronic/exonic sequence. The band marked with a white asterisk in *tmc5* morphants showed similar levels to wild type embryos; however, these embryos also displayed a prominent mis-spliced product.

Supplementary Fig. S7. The FIGs form a distinct collection compared to previous motile cilia gene screens. (A) Comparing the FIGs to genes expressed early or late after induction of ciliogenesis in mouse multiciliated tracheal epithelial cells (MTEC) reveals an overlap of just 135 genes (23.6% of the FIGs). (B) There are 55 common genes between the FIGs and genes upregulated by the multiciliated cell fate determinant Multicilin (9.6% of the FIGs). (C) Comparing the FIGs to previously

identified Foxj1 target genes reveals an overlap of just 60 genes (10.5% of the FIGs). (D) There are 111 common genes between the FIGs and genes upregulated by another ciliary transcription factor, Rfx2 (19.4% of the FIGs). (E) An assessment of the various motile cilia screens with respect to the identification of the 26 known PCD genes. Black bars represent the percentage of the known PCD genes present in each screen, while the area of the circle is proportional to the number of genes identified in each screen.

Supplementary Movie 1. Immotile cilia on muscle cells of a 24 hpf *Tg(actb2::arl13b-GFP)* embryo

Transgenic zebrafish expressing the Arl13b protein tagged with GFP contain GFP labelled primary cilia. Primary cilia from muscle cells showed no movement when imaged over time.

Supplementary Movie 2. Motile cilia on muscle cells of a 24 hpf *Tg(actb2::arl13b-GFP)* embryo overexpressing Foxj1

When double transgenic *Tg(actb2::arl13b-GFP; hsp70::foxj1a)* embryos were heat shocked to induce Foxj1 overexpression, labelled cilia of muscle cells showed rhythmic beating when imaged over time.

Supplementary Movie 3. KV motile cilia in a wild type embryo expressing Arl13b-GFP

A sample movie of KV cilia motility in a wild type embryo at 14-15 hpf with GFP-labelled cilia. Video acquisition was at 333 frames/s and playback is at 11.8 frames/s.

Supplementary Movie 4. KV motile cilia in a control morpholino-injected embryo expressing Arl13b-GFP

A sample movie of KV cilia motility in a control morpholino-injected embryo at 14-15 hpf with GFP-labelled cilia. Video acquisition was at 333 frames/s and playback is at 11.8 frames/s.

Supplementary Movie 5. KV motile cilia in a *plcxd2* morpholino-injected embryo expressing Arl13b-GFP

A sample movie of KV cilia motility in a *plcxd2* morpholino-injected embryo at 14-15 hpf with GFP-labelled cilia. Video acquisition was at 282 frames/s and playback is at 10.0 frames/s.

Supplementary Movie 6. KV motile cilia in a *zbbx* morpholino-injected embryo expressing Arl13b-GFP

A sample movie of KV cilia motility in a *zbbx* morpholino-injected embryo at 14-15 hpf with GFP-labelled cilia. Video acquisition was at 282 frames/s and playback is at 10.0 frames/s.

Supplementary Movie 7. KV motile cilia in a *lace1b* morpholino-injected embryo expressing Arl13b-GFP

A sample movie of KV cilia motility in a *lace1b* morpholino-injected embryo at 14-15 hpf with GFP-labelled cilia. Video acquisition was at 321 frames/s and playback is at 11.4 frames/s.

Supplementary Movie 8. KV motile cilia in a *tp53bp2* morpholino-injected embryo expressing Arl13b-GFP

A sample movie of KV cilia motility in a *tp53bp2* morpholino-injected embryo at 14-15 hpf with GFP-labelled cilia. Video acquisition was at 334 frames/s and playback is at 11.8 frames/s.

Supplementary Movie 9. KV motile cilia in a *kcnip1a* morpholino-injected embryo expressing Arl13b-GFP

A sample movie of KV cilia motility in a *kcnip1a* morpholino-injected embryo at 14-15 hpf with GFP-labelled cilia. Video acquisition was at 159 frames/s and playback is at 5.6 frames/s.

Supplementary Movie 10. KV motile cilia in an *aftphb* morpholino-injected embryo expressing Arl13b-GFP

A sample movie of KV cilia motility in an *aftphb* morpholino-injected embryo at 14-15 hpf with GFP-labelled cilia. Video acquisition was at 334 frames/s and playback is at 11.8 frames/s.

Supplementary Movie 11. KV motile cilia in a *tmc5* morpholino-injected embryo expressing Arl13b-GFP

A sample movie of KV cilia motility in a *tmc5* morpholino-injected embryo at 14-15 hpf with GFP-labelled cilia. Video acquisition was at 378 frames/s and playback is at 13.4 frames/s.

Supplementary Movie 12. KV motile cilia in a *spa17* morpholino-injected embryo expressing Arl13b-GFP

A sample movie of KV cilia motility in a *spa17* morpholino-injected embryo at 14-15 hpf with GFP-labelled cilia. Video acquisition was at 484 frames/s and playback is at 17.1 frames/s.

Supplementary Movie 13. KV motile cilia in an *arhgef18b* morpholino-injected embryo expressing Arl13b-GFP

A sample movie of KV cilia motility in an *arhgef18b* morpholino-injected embryo at 14-15 hpf with GFP-labelled cilia. Video acquisition was at 156 frames/s and playback is at 5.5 frames/s.

Supplementary Movie 14. KV motile cilia in an *ak9* morpholino-injected embryo expressing Arl13b-GFP

A sample movie of KV cilia motility in an *ak9* morpholino-injected embryo at 14-15 hpf with GFP-labelled cilia. Video acquisition was at 139 frames/s and playback is at 4.9 frames/s.

Supplementary Table S1. Comparison between the zebrafish FIGs and the mammalian and *Chlamydomonas* genomes, and previous cilia screens. The 596 zebrafish genes up-regulated at least 2-fold by Foxj1 are listed. Given are the Ensembl Gene ID, chromosome, gene start and gene end. The zebrafish gene symbol and version number (if there are multiple genes with the same gene symbol) along with the source of the annotation (either ZFIN, UniProtKB Gene Name, HGNC Symbol, Clone-based (Ensembl) or UniGene) are also included. Vertebrate ortholog Ensembl Gene ID (Human or Mouse) along with the source of the ortholog prediction, the gene symbol, description of the ortholog and the annotation source for the vertebrate gene are listed. In addition, the microarray data are included consisting of the sequence of the representative probe, the average fold increase of all of the probes representing the gene, and the p-value indicating the significance of the measurement (Student's t-test with multiple hypothesis correction). The next two columns indicate whether the gene is present in the cilia proteome (http://v3.ciliaproteome.org/cgi-bin/) or the ciliome (www.ciliome.com). The final

column indicates whether or not the gene has an ortholog in the *Chlamydomonas* genome. Foxj1, highlighted in green, is the positive control in the study.

Supplementary Table S2. A large number of known ciliogenic and ciliopathic genes are found amongst the FIGs. The 83 FIGs that are known to be ciliogenic or ciliopathic in a model organism are listed. Gene annotation is included, starting with the zebrafish Ensembl Gene ID, the zebrafish gene symbol and version number, and the vertebrate ortholog Ensembl Gene ID, gene symbol and description. If the gene has been previously implicated in a human ciliopathy, the Human Ciliopathy and OMIM number is listed. A reference describing the ciliogenic or ciliopathic function of the gene is provided. Genes which cause human ciliopathies are highlighted red.

Supplementary Table S3. Fifty randomly selected FIGs with detailed expression, localization and knockdown data. The first eight columns of each of the five sheets contain, in order: the designated gene name, the zebrafish Ensembl Gene ID, Gene Symbol and version. This is followed by the verterbrate ortholog Ensembl Gene ID, Symbol and Description. An internal CG code is also given (CG0XX) for reference. Sheet 1 (Zebrafish qPCR) includes the zebrafish qPCR forward primer name and sequence, followed by the qPCR reverse primer name and sequence for each gene. The last three columns of this sheet contain the fold change increase in Foxj1 overexpressed embryos relative to wild type, the standard error of the mean (SEM) for each fold change, and the p-value when a t-test is performed comparing the gene

expression levels in the Foxj1 overexpressed embryos to the levels in wild type. All of the data were normalized against expression levels of a housekeeping gene, *rplp0*, and *actb1* levels were checked as a negative control (highlighted in green). Sheet 2 (RACE and Localization Results) lists the results of the RACE and sequence analysis along with the protein localization results. Controls include embryos injected with mRNA encoding GFP alone and immunofluorescence analysis of wild type, uninjected embryos (highlighted in green). Sheet 3 (Morpholino Information) lists the morpholino version (an internal label), the sequence of each antisense morpholino (5′ to 3′), the transcript against which the morpholino was designed, and the exon/intron junction of the canonical transcript which the morpholino targets. The next two columns contain the primer names and sequences for the oligonucleotides used to test the mis-splicing for each morpholino (forward and reverse primers, respectively). Finally, the amount of morpholino (in picomoles) injected into each embryo for phenotypic analysis is given along with the results of the PCR and sequencing. Sheet 4 (Morpholino Phenotypes) lists the counts for each of the phenotypes assayed during the morpholino analysis. The numbers of affected, normal, and total embryos are given for each phenotype, in addition to the percentage of affected embryos and the p-value of the Fisher's exact test comparing the morpholino-injected embryos with wild type embryos. Phenotypes analyzed include: curved body axis (CBA), otolith counts, hydrocephalus, kidney cysts and left-right asymmetry. The standard control morpholino-injected and wild type embryo analyses are highlighted in green. Sheet 5 (Cilia Length and Motility)

includes average length measurement of pronephric cilia along with standard deviations and p-values. In addition, KV cilia motility data is provided for the ten assayed morpholinos, including average percent motility and average beat frequency, standard deviations and p-values. Control morpholino-injected embryos and wild type embryos are reported as controls (green). Sheet 6 (Human qPCR) includes the human qPCR forward primer name and sequence, followed by the qPCR reverse primer name and sequence for each gene. The next column contains the results of the expression analysis by qPCR for human tissues. The final three columns contain the fold change increase in human brain, lung, testis or heart relative to skeletal muscle, the standard error of the mean (SEM) for each fold change and the p-value when a t-test was performed comparing the gene expression levels in the tissue in question relative to muscle. All of the data were normalized against expression levels of a housekeeping gene, *ACTB*, while *GAPDH* levels were checked as a negative control (both highlighted in green).

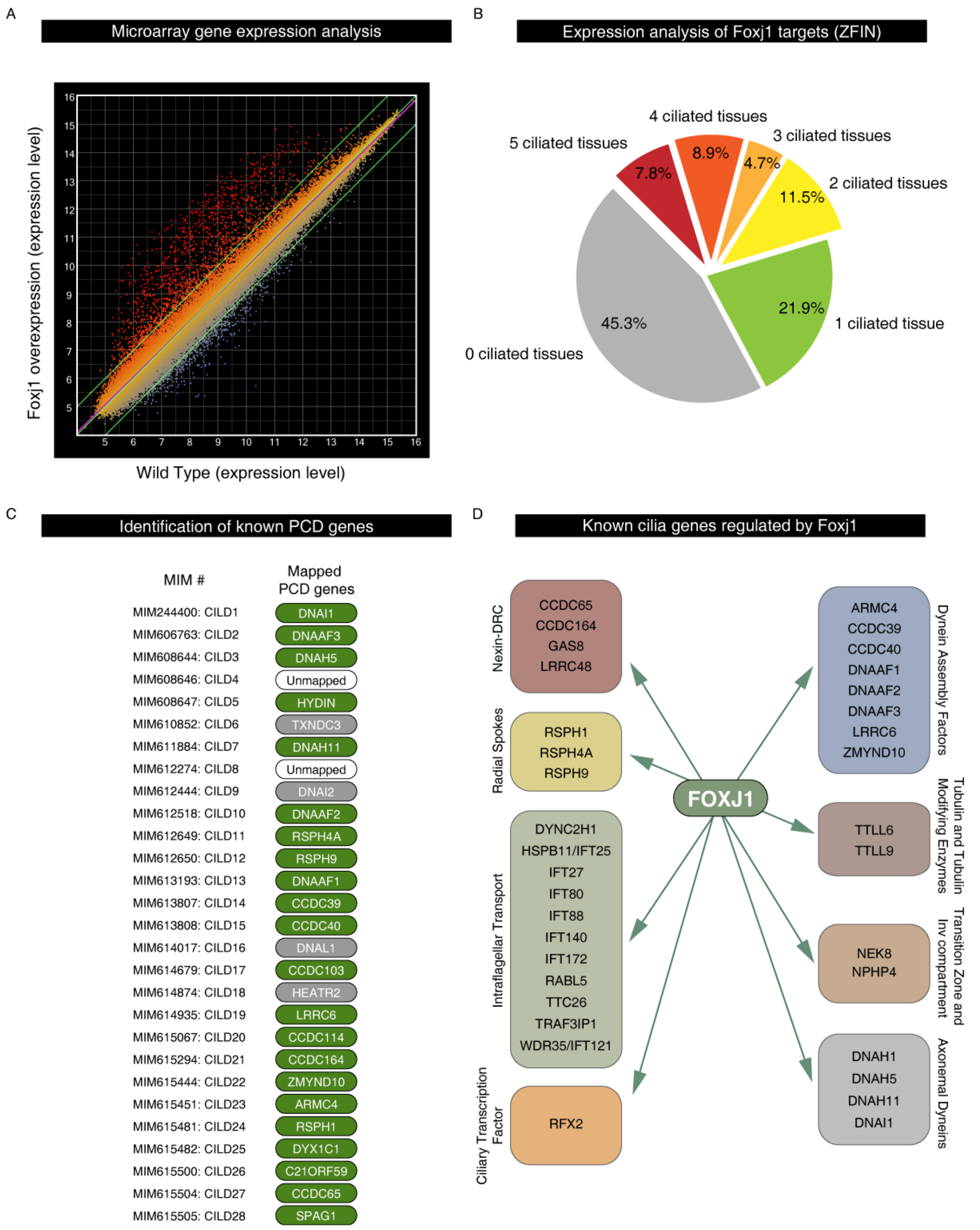

Supplementary Fig. S1

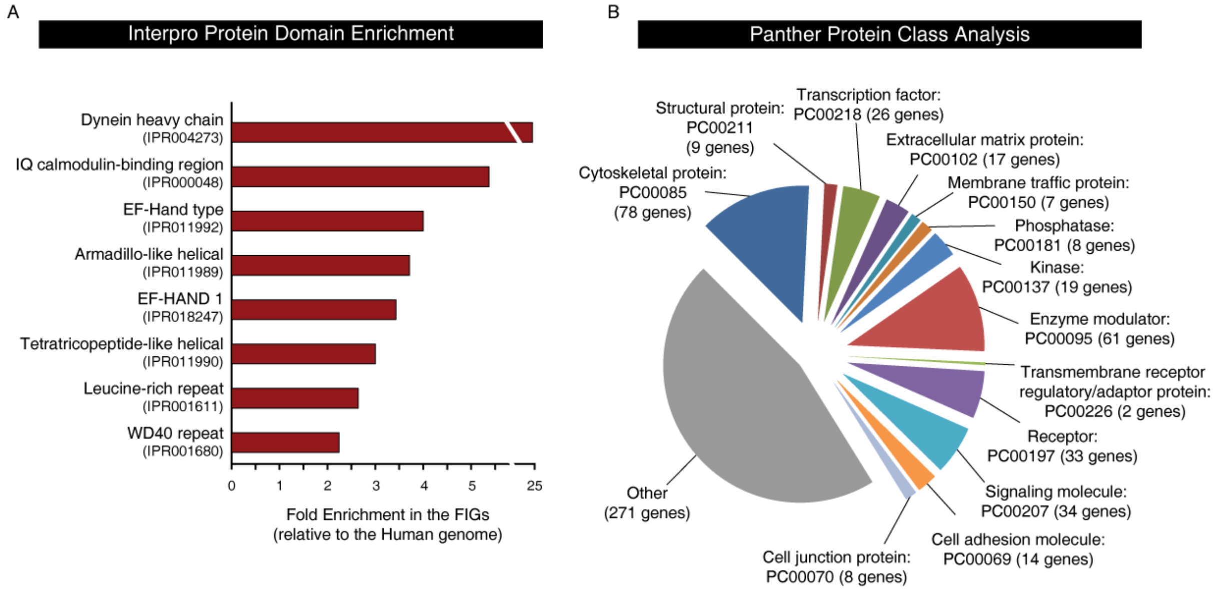

Supplementary Fig. S2

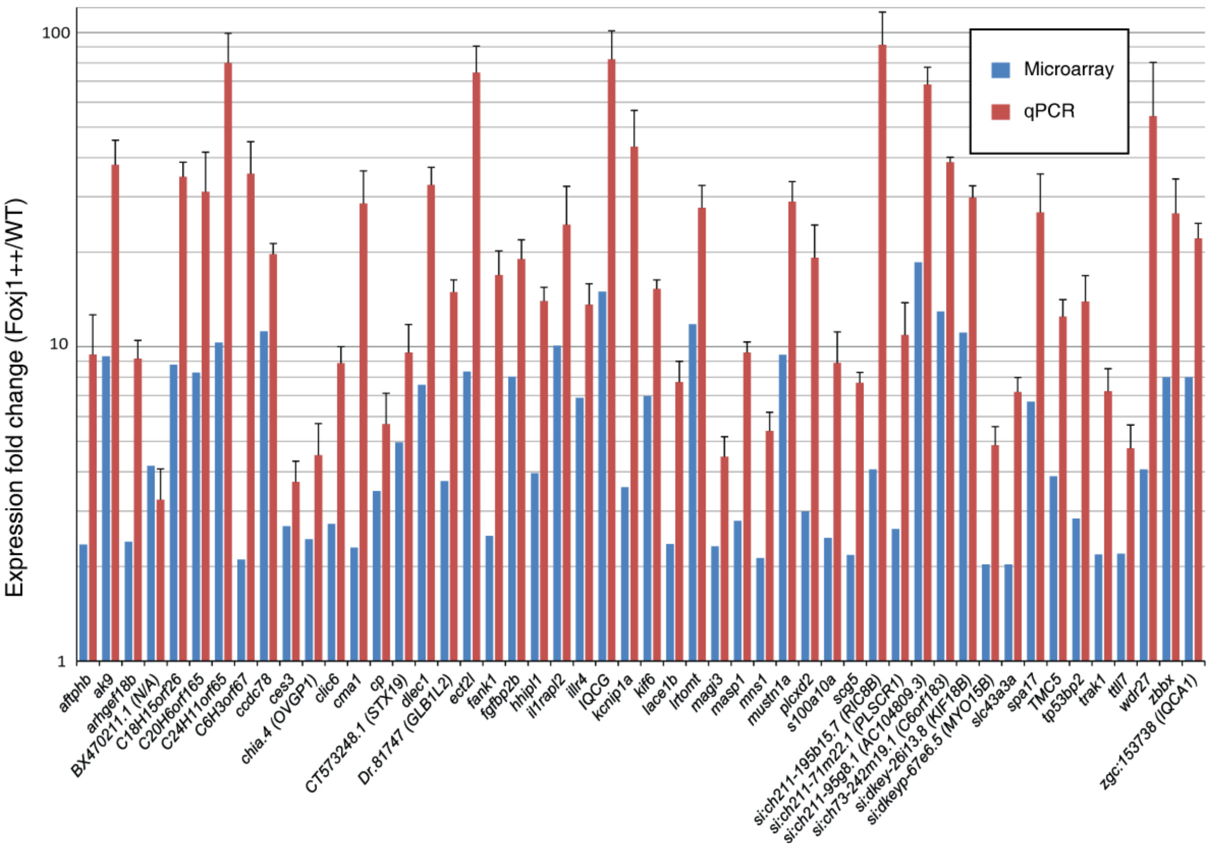

Supplementary Fig. S3

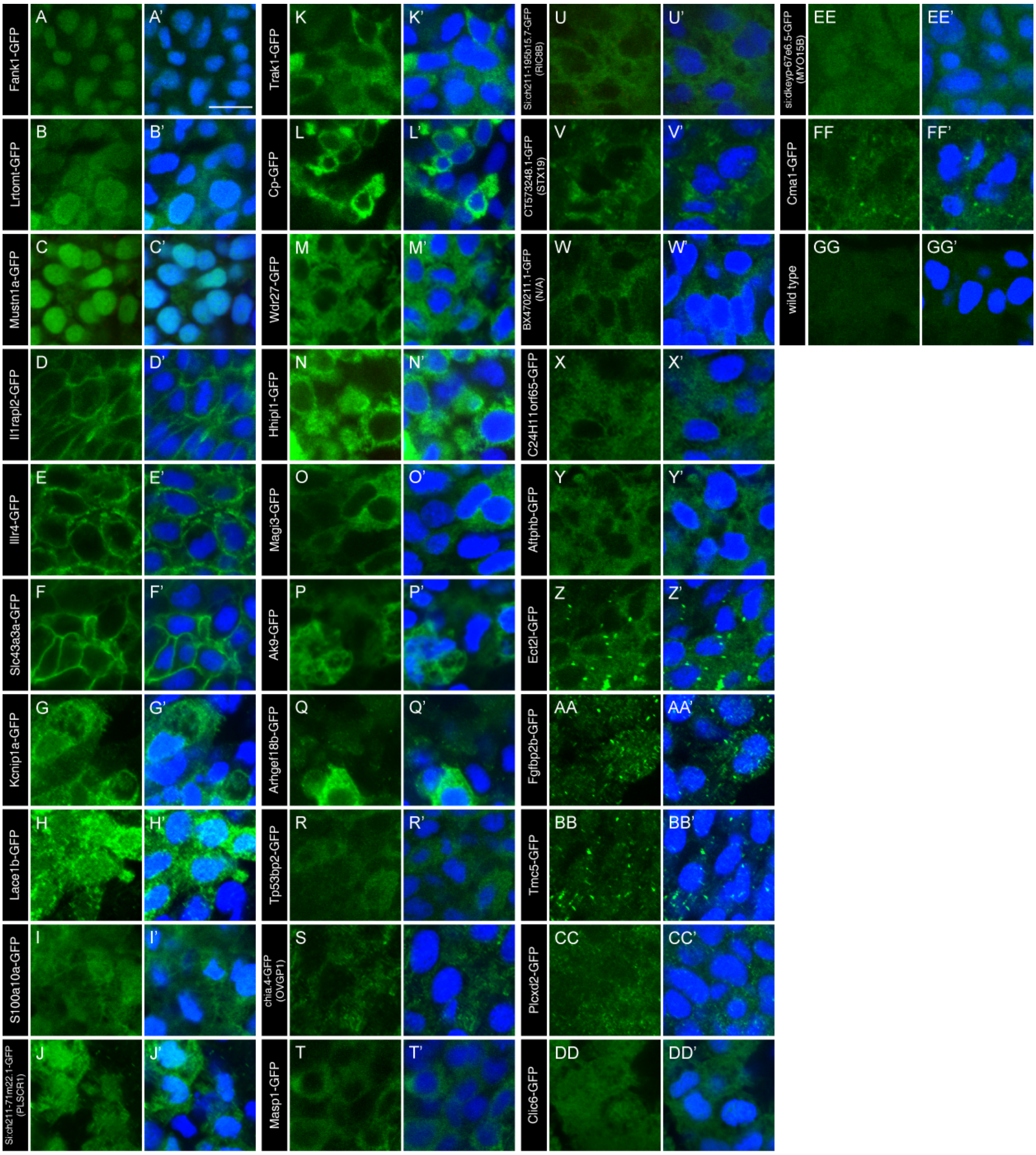

Supplementary Fig. S4

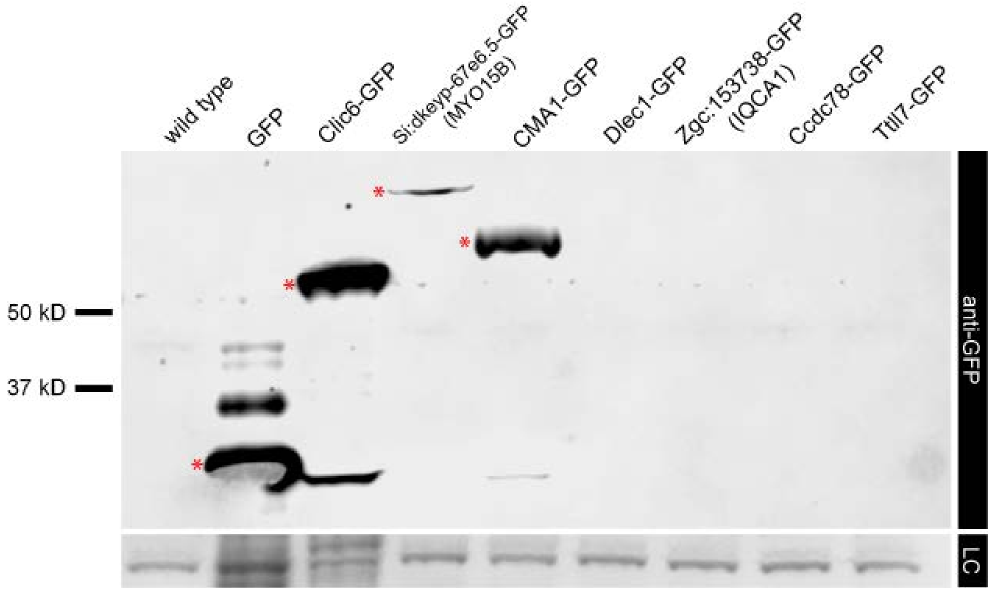

Supplementary Fig. S5

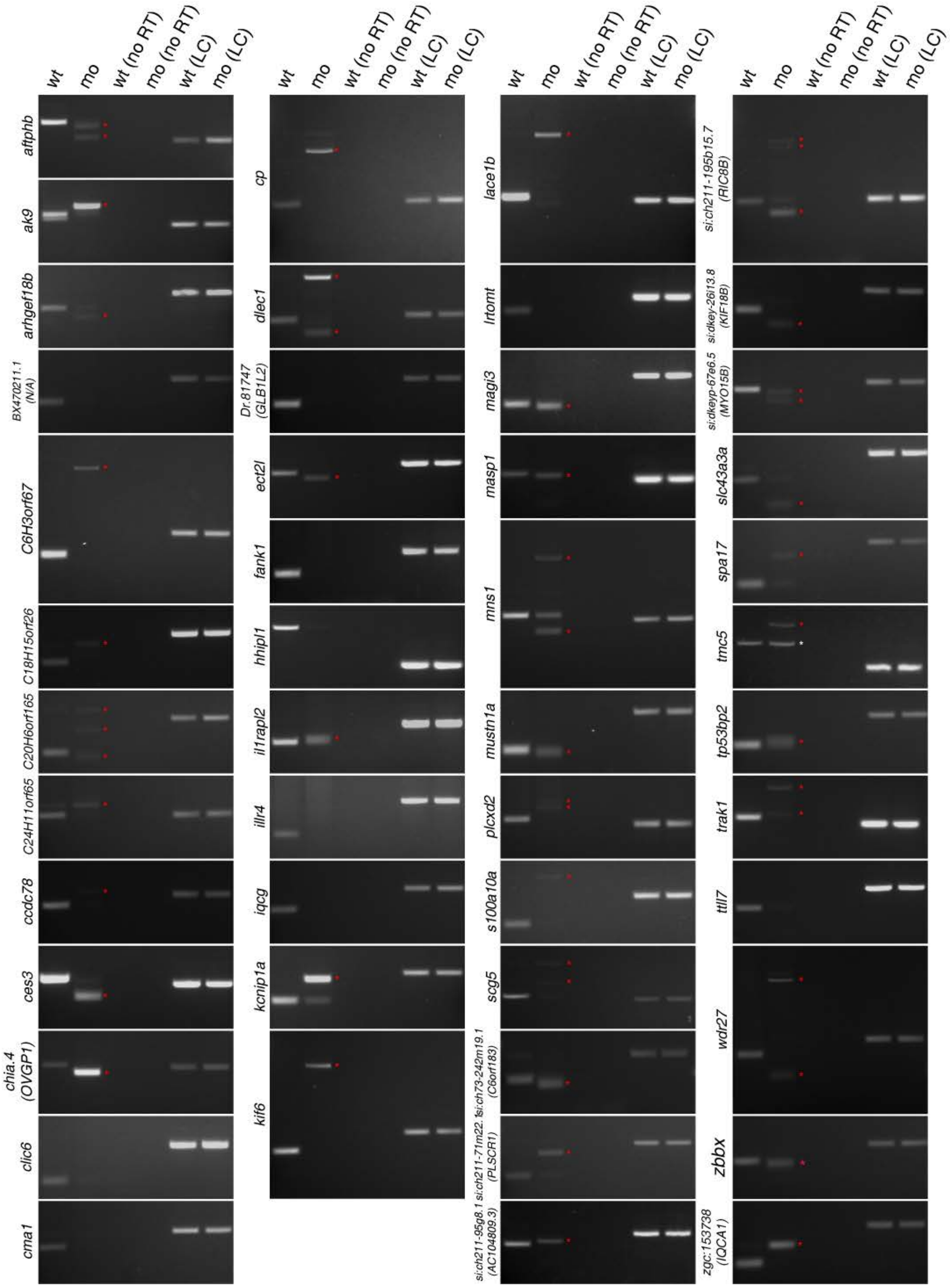

Supplementary Fig. S6

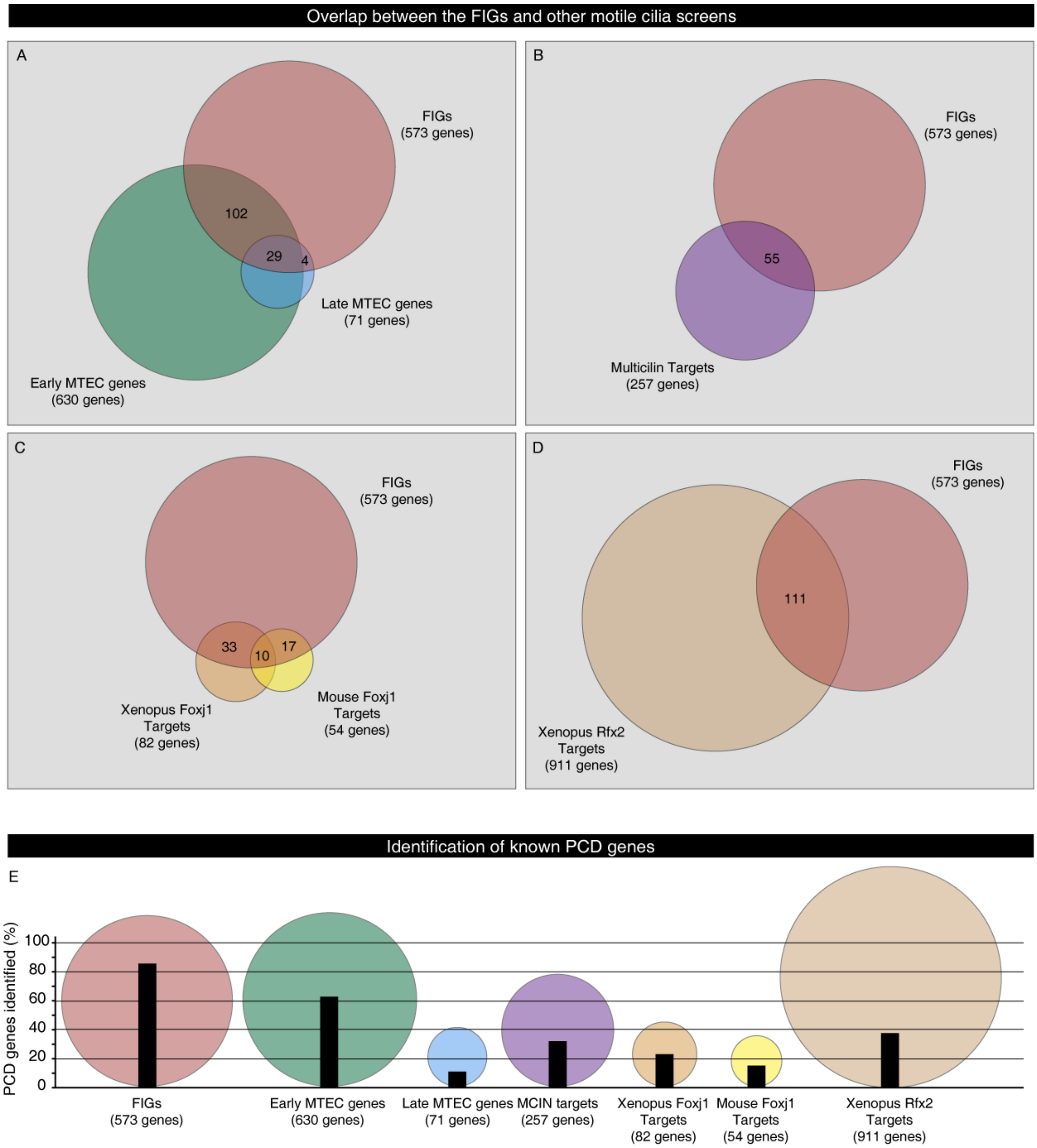

Supplementary Fig. S7

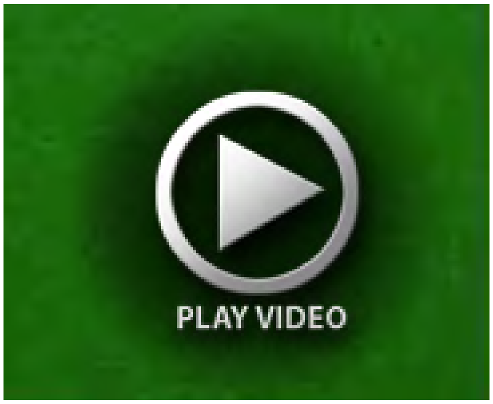

Movie 1.

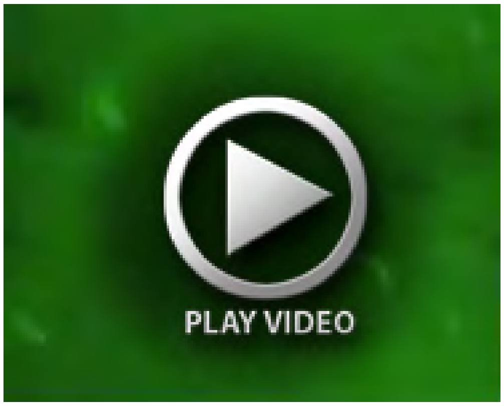

Movie 2.

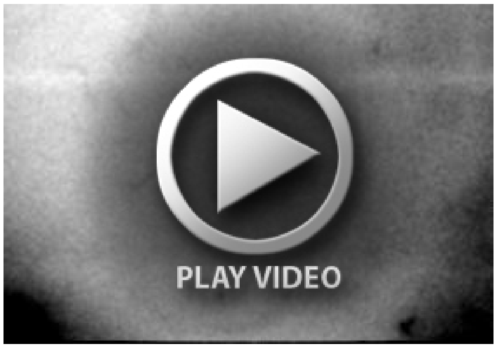

Movie 3.

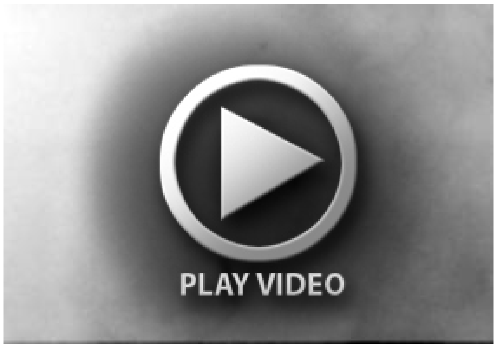

Movie 4.

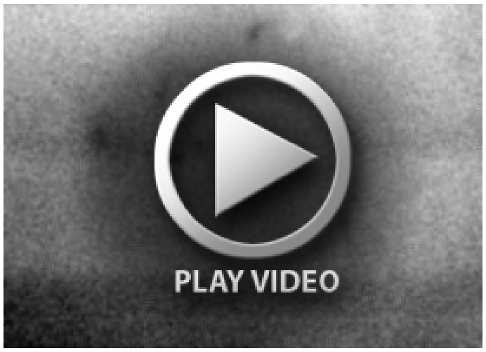

Movie 5.

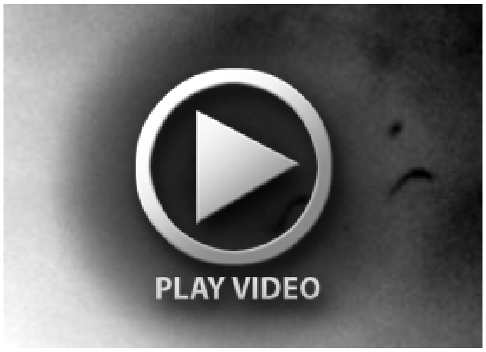

Movie 6.

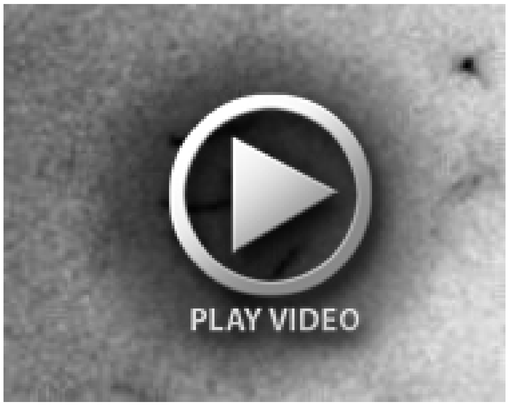

Movie 7.

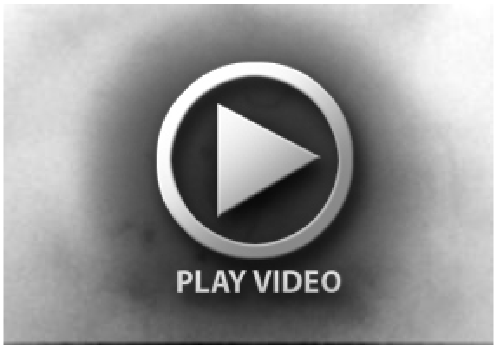

Movie 8v.

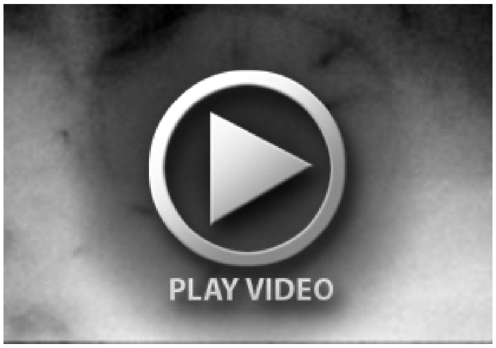

Movie 9.

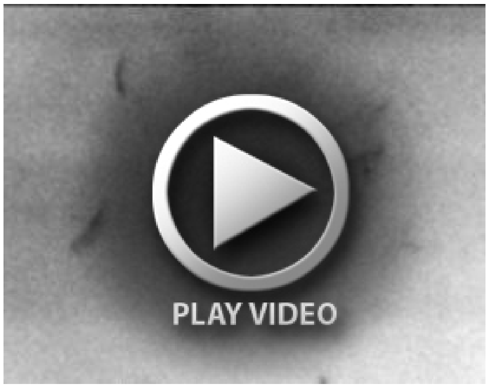

Movie 10.

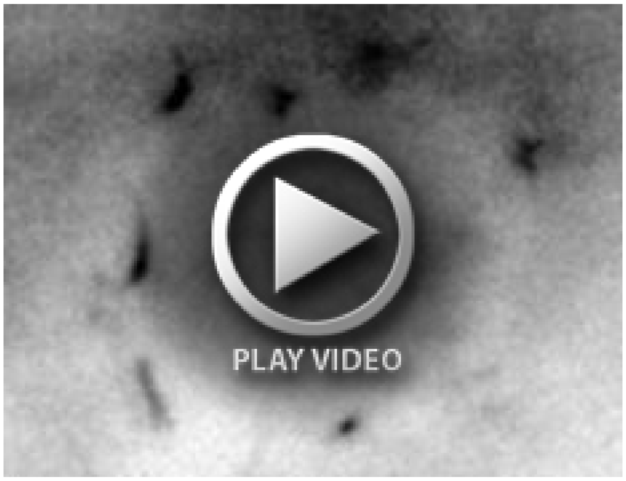

Movie 11.

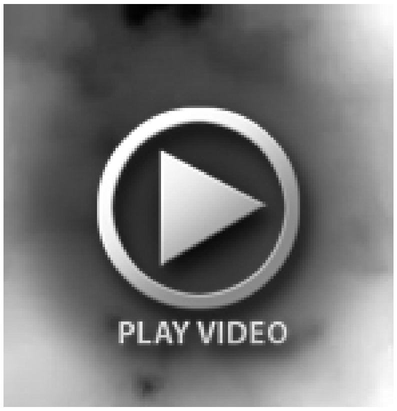

Movie 12.

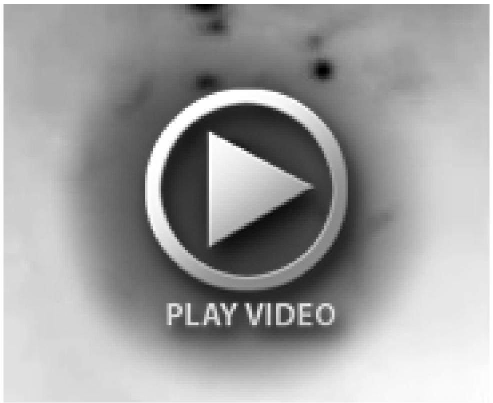

Movie 13.

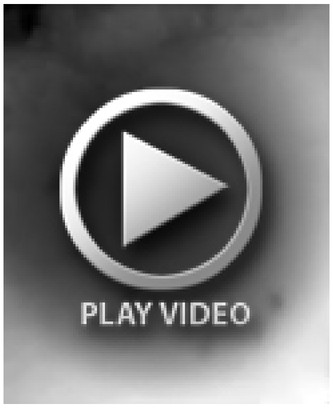

Movie 14.

Download Table S1

Download Table S2

Download Table S3
